# Beginning in Multiple Midst: Living Alongside in Narrative Inquiry

**DOI:** 10.1177/10778004241308179

**Published:** 2025-01-06

**Authors:** Bodil H. Blix, Pamela Steeves, Vera Caine, Jean Clandinin

**Affiliations:** 1 UiT The Arctic University of Norway, Tromso, Norway; 2 University of Alberta, Edmonton, Canada; 3 University of Victoria, British Columbia, Canada

**Keywords:** narrative inquiry, relational, refugee families, methodological approaches

## Abstract

Narrative inquiry is often represented by using fragments of field texts. Drawing on a 4-year study alongside refugee families from Syria, we show the importance of acknowledging the multiple and nested contexts within which field texts are situated. We illustrate how each moment is reflective of being in the midst—of social, institutional, and cultural narratives, formula stories, multiple relationships, domestic details, and silences. Attending to living in the midst allows us to contemplate perplexities amid a plurality of experiences that are embedded and understood as part of living within unfolding lives and relationships.

*In preparation for teaching a guest doctoral seminar, I (Vera) read the assigned texts provided to the students about narrative inquiry. I was surprised that examples of narrative inquiry began with isolated fragments of stories from one of our studies. While the fragments were appropriately cited, I saw how the narrow focus erased the relational work necessary for understanding. There was no sense of how we, as narrative inquirers, engaged in the field, became part of relationships, and lived in the midst of ongoing lives. Later, alongside Jean, Pam, and Bodil, I wondered how to make more evident the relational work of living alongside participants in narrative inquiry.*

For us, narrative inquiry is based on the understanding of experience as always evolving, always in the making, and always being shaped and reshaped over time (Caine et al., 2022). As a relational methodology, “we intentionally come into relation with participants, and we, as inquirers, think narratively about our experiences, about our participants’ experiences, and about those experiences that become visible as we live alongside” (Clandinin, 2013, p. 23). Narrative inquiry is not only about listening to stories that people tell *about* experiences. Narrative inquiry is also about living alongside. Clandinin and Connelly (2000) wrote that there are two starting points for narrative inquiry: telling stories and living stories of experience. When we engage with participants in telling stories, field texts are mainly conversation transcripts, as well as artwork, photographs, both purpose-taken and archival, annals and chronicles, and memory box artifacts. In the second starting point, that is, living stories, we come alongside participants over time and in different places and contexts. With both starting points, we engage in ongoing inquiry with participants’ lives and with our own lives. In narrative inquiry relationships in which we live alongside experience with participants, we see the unfolding enfolding nature of experience more easily. It is in such relationships that we see that participants, and we, live experiences as much as we tell them in words (Neumann, 1997).

In this paper, we inquire into what it means to find ourselves and participants in the midst of living unfolding relationships, in the midst of multiple stories and domestic details, as well as in the midst of silences. Drawing on the work of American feminist pragmatist Jane Addams (1902), we contemplate notions of humility and perplexity as we consider what it means to be in the midst of living alongside participants. Addams (1902) lived and worked at Hull House in Chicago when the burgeoning immigrant population faced poverty brought about by the industrial revolution. Addams did not turn away from the ethical dilemmas but, instead, set out to develop a conception of social ethics “not attained by travelling a sequestered byway, but by mixing on the thronged and common road where all must turn out for one another, and at least see the size of one another’s burdens” (p. 3). As Addams experienced the everyday lives of immigrants of diverse ethnic backgrounds and of degrees of poverty and distress, she recognized the complexity inherent in composing relational spaces in situations of unequal power and privilege. She adopted a stance of humility in her interactions with her Hull House neighbors which encouraged openness, reimagination, and growth. As Addams (1902) wrote, “we have learned as common knowledge that much of the insensibility and hardness of the world is due to a lack of imagination which prevents a realization of the experiences of other people” (p. 4). In narrative inquiry, we become part of the lives of participants and they become part of ours (Clandinin, 2013). In living alongside, when we find ourselves in the midst of their and our lives, we can better understand the complexities of lives and the spaces that we co-compose.

We draw upon a narrative inquiry with the experiences of families from Syria who sought refuge in Canada in the early 21st century. Addams’s (1902) ideas helped us see that coming alongside mothers and children from Syria was a commitment to travel together on a common road. Coming alongside in everyday ways to co-compose relationships allowed us to actively participate in the lives of mothers and their children. Through active participation, we were awake to the ways our presence alongside them enriched our listening and our noticing. Hamington (2001) wrote of Addams as engaging in a kind of active listening. This notion that she was both consciously and unconsciously immersed in the circumstances of her immigrant neighbors’ lives resonated with us.

We arrive at the two-story building located in a strip mall in northeastern Edmonton for a regular Friday morning commitment. Vera still remembers the first visit and how hard it was to find the place. Jean, Hiroko, and Vera often travel together. Today we are joined by Gillian and Heather. Carefully taking bags of fruit and buns from the back of Vera’s car, we climb the stairs to the gathering room. We’re greeted by two members with responsibility for the Friday morning English language classes for newly arrived mothers from Syria. The mothers bring their preschool children as care is provided in a playroom while the mothers are in class. We’ve been coming now for about three months.

We take off our coats and boots and take the snacks to the table in the main classroom. Mothers and their children begin to arrive and the noise level rises. We greet each of the mothers with smiles and hellos and a few of them hug us as they now know us. We help children take off their boots and snow clothes. When enough mothers have arrived, we are all invited to the big table where the English as a Second Language instructor is setting out worksheets and supplies for the day’s lesson. At first we felt that we might have caused (dis)ease for the instructor by our presence. When we offered our help by sitting alongside the mothers, she began to include us in her lessons and asked us to work with the mothers as they did the assigned tasks. On this day, Gillian, who had brought her own child, headed into the children’s playroom to work with the caregivers.

After about 60 minutes it is time for snacks and the children rejoin their mothers. As snacks are taken from the snack table, we and the mothers find spaces on floor cushions or chairs in the carpeted area and conversations about lives in motion open up. Both Arabic and English are spoken and the blend of language and laughter emanates from the room. By now the mothers ask us questions about our lives and questions about what they are puzzled by in their own lives. Often we look for others who can translate words; sometimes we just listen to the sounds and gestures as we notice gentle stroking of a child’s head. These are, for us, signs that we and the women are at ease.

This account of our work in the Friday morning sessions is a moment from the midst of a narrative inquiry study, a moment that is reflective of being in the midst—of multiple relationships, stories, domestic details, and in the midst of silences. We are also in the midst of larger social, institutional, and cultural narratives, narratives that often are not visible in the immediate living. We, alongside community members, carefully planned this narrative inquiry study to better understand the experiences of families who arrived from Syria with preschool-aged children. We were interested in the families’ early encounters with school systems. Our interest was shaped by our engagement with schools, ideas of diversity and inclusion, but also our involvement in responding to the influx of Syrian refugees in Canada and Edmonton specifically.

## In the Midst of Narratives of What Counts as “Good” Research

Narrative inquiry is lived and conducted among narratives about research quality. Since we resist conceptualizations of the researcher as “listener,” ‘collector,’ and “analyst,” and the participant as “teller” based on the Western scientific ideal of the researcher as an “objective observer,” we often have to explain what we do. These dominant narratives of “good research” frame and restrict how we can compose proposals for funding and interact with ethics committees. We have heard echoes of these narratives in numerous peer review reports and in feedback from colleagues throughout the years—critique based on the assumption that narrative inquiry is the study of personal experience without attention to the wider social, political, material, and cultural contexts shaping experience. Such experiences remind us that narrative inquiry is radically at odds with prevailing scientific ideals of objectivity, consistency, and replicability because the phenomenon under study, the participants, and the inquirer are all changing throughout the inquiry process. It is in the midst of this space composed and restricted by broader narratives, participants, and we, must create a new space for the living and co-composition of reconfigured and reimagined narratives of research. And it is in the midst of spaces we, as researchers, are part of the phenomenon being studied.

Our work on reconceptualizing who we see ourselves to be as researchers has been discussed in terms of seeing ourselves in the metaphoric parade that is under study (Clandinin & Connelly, 2000). That is, we understand that we are complicit in the dominant narratives that shape the situations in which we find ourselves as well in shaping the immediate relational events under study. We see that the ways we analyze and represent events, people and places under study shape both what we make visible and what we hide in our work. Making ourselves visible in these ways is central to the ontological and epistemological claims we make. We are reminded of how easy it is for others to only pull forward fragments of studies, while losing a sense of the long-term engagement and commitments to the research relationships we hold.

## In the Midst of Formula Stories and Dominant Narratives

As narrative inquirers we know that people live and tell their stories in the midst of broader narratives. Clandinin (2013) noted that the stories we tell “reflect the prevailing theories about ‘possible lives’ that are part of one’s culture” (p. 191). We know that both participants’ and our own stories are framed and shaped by stories about “us” and “them” that may impose limitations on our imaginative space. Loseke (2007) used the term “formula stories” about “collective representations of disembodied types of actors . . . producing such categorical identities associated with families, gender, age, religion, and citizenship” (p. 663). Such “formula stories” represent a turning away from experience rather than a staying with experience. These stories enable the fragmentation of identities and are often connected to fixed identities. They rarely allow us to see that participants and we live in the midst and are always composing identities within multiple and often competing narratives.

While “formula stories” point to disembodied categories, we also encounter dominant narratives. Dominant narratives are a reflection of particular social interests and ideologies. We are familiar with dominant narratives circulating about refugees, refugee families, Muslim women, and even the framing of their lives as a “refugee crisis.” Dina Nayeri (2019) in her book, *The Ungrateful Refugee: What Immigrants Never Tell You* made visible some dominant narratives as she inquired into her own experiences, and those of other refugees, that shaped their stories of being refugees as ones in which they composed, lived, and continued to tell stories of gratefulness, humility, and compliance, rather than stories shaped by regret and resistance. As Nayeri pointed out, if refugees chose to tell stories with other plotlines that emerged from their experiences, they risked being storied as ungrateful, demanding, opportunistic, and perhaps even as a threat to society. As narrative inquirers, we were awake to these broader social and cultural narratives of “us” and “them,” fueled by representations of what refugee women from Syria were lacking in terms of skills and resources. As we continually worked to push back against these dominant narratives, we, carefully and slowly, co-composed spaces in which other stories could be lived and told and new experiences could unfold. We paid close attention to possibilities that resisted both the reinforcement of dominant narratives, as well as the formula stories that erased the complexities of who we are and that we are always living in the midst.

## In the Midst of Multiple Unfolding Relationships

Vera has a long-standing relationship with the Multicultural Health Brokers in the city of Edmonton where our study was located. Through Vera’s work with a student with a strong interest and commitment to the mental health of refugee populations, Vera became more involved in thinking about ways to respond to the health and social needs of refugees from Syria. Agencies came together to form a response to the arrival of many refugees around 2015. One such place was the monthly meetings of Coalition of Social Inclusion (CoSI). As part of the coalition, there were many dialogues about the meaning of social inclusion. Through these dialogues among people from community agencies, people with experiences of being refugees, government employees, and health care professionals, our study evolved. The study was also shaped by Jean and Heather’s long-term commitment to people whose lives were shaped by schools and included children, families, teachers, and administrators. Each research team member had lived and worked in multiple communities with good intentions for many years.

In our proposal for funding, we wrote that participating in the Friday morning groups was our way of gaining entry into the refugee community and to finding participants for our study. However, we realized over time that our Friday morning participation was neither our entry point, nor our destination. The Friday morning gatherings amongst Syrian women and their children became a place to build relationships, to live amidst ongoing joys and hardships, amidst celebrations, and most of all, amidst silences and contemplations. Many Friday gatherings might have been described as ordinary in the lives of refugees, places that allowed them to access resources, to meet others, and to gain the support that was necessary for a sense of social inclusion. Many of the Friday morning gatherings for us, as researchers, could also be described as ordinary, not much was happening and for researchers used to gathering data, it might have seemed unproductive. Yet, it was also extraordinary for all of us in that it provided a space to make friends, to slow down and dwell in the silences and gestures, in the tears and laughter that were present in the children’s play, and in the longing of children to be close to their mothers. Each Friday became a way to shape our relationships with possible participants, it became a way to imagine a way of entering people’s lives slowly and with intention.

As we wrote our field notes and thought about who we were in the Friday morning gatherings we were startled to realize that we were not there just as researchers, although we were always present as researchers. But it was our presence as mothers, grandmothers, daughters, sisters, teachers, nurses, and administrators that became important to seeing ourselves as people among people whose life experiences were markedly different from ours. We were all people composing our lives. We were reminded that the spaces where we meet are always “spaces of belonging for both researchers and participants; spaces that are marked always by ethics and attitudes of openness, mutual vulnerability, reciprocity, and care” (Clandinin & Caine, 2013, p. 169).

While participating in the Friday morning gatherings for well over a year allowed us to meet participants we lived alongside, it was also a space that opened considerations of our uncertainty, emotions, and puzzlement as narrative inquirers. This co-composition also shaped our being in relation, where reciprocity and recognition were entangled (Blix et al., 2023).

## In the Midst of Living Alongside: Domestic Details

It was on the Friday mornings, spent in the language classes with the mothers and children, that we became most attentive to how we were enfolded in the families’ lives in ways that allowed us to come alongside. Sometime before the break for the December holiday, the mothers and staff decided to organize a party for the Friday morning time. We offered to bring food, much like we had done every Friday—perhaps an ordinary gesture by now. Yet, when we arrived that day, we found ourselves amidst a larger gathering of women and children, dressed in beautiful celebratory dresses. The table where we gathered for classes was laden with a multitude of traditional Syrian food and music that was unfamiliar to our ears. Music played on a phone over a speaker and a table was set up where everyone was invited to receive henna designs on their hands. We mingled amongst everyone, conscious of our casual clothes and lack of familiarity with the celebration. One of the women eventually asked Jean to dance, all while telling her we were celebrating a wedding. The puzzlement on our faces must have spoken volumes, eventually we realized that this was an enactment of a wedding. However, it was, the woman assured us a replica of what really happened at a wedding, particularly when the women gathered without the men. The children were delighted, running around, some of the older children missed school to be able to attend. The joy was palpable, we all shared food, danced and laughed. The children were given instructions about wedding ceremonies and behaviours. While the women took care to make sure we were included by speaking some English and inviting us to the henna painting and dancing and food, the language was mostly Arabic and the women and children were clearly enjoying the wedding celebration. When the morning ended and husbands began to arrive, the women carefully apportioned the leftover food for each family to take home and there were hugs and goodbyes as the party ended.

Memories of what happened that day stayed with us. It opened up new possibilities to think with our experiences and the experiences of participants. The concept of domestic detail (Child, 1972, as cited in Pratt, 2002), was described as

the particulars of the women’s lives and relations that occurred in a particular location. More than a story of place, then, meaning becomes a story of the domestic context where “domestic” is taken in its oldest sense of having to do with where one lives, one’s home and relations (p. 235).

As we worked to understand our experiences of the celebratory Friday morning, the concept of domestic detail seemed particularly fitting. We recognized that something was happening that was linked with where the women lived, their homes, and their relations. We talked about the wedding celebration on subsequent Friday mornings and each time it brought smiles to people’s faces. It also brought out ordinary stories of meeting at people’s homes for coffee, of gathering during the days with family members, neighbors, and friends in Syria. Sometimes memories were evoked as we smelled coffee brewing or tasted the first sip during our morning break. The coffee in Syria always seemed so much better. Using Child’s idea, the meaning of the event was rooted in the women’s domestic contexts.

Being confused and uncertain as we encountered the “wedding party” that day, we might have shaped a curt dismissal of all that was going on but instead we embraced our perplexity as an opportunity to think anew. Addams showed that perplexity can reveal “a rupture with conventional attitudes, beliefs, and practices, something that cannot be resolved without developing a new understanding of the situation and calling into question received values” (Siegfried, 2002, p. xxii). We are also reminded of deCruz (2021) who wrote:

[T]here are situations where our everyday routines break down, where, as John Dewey (1939, p. 33) put it “there is something the matter”; this is a situation where “there is something lacking, wanting, in the existing situation as it stands, an absence which produces conflict in the elements that do exist.” When things run smoothly, we are not motivated to rethink our routines. When it is no longer possible to continue as we are, we have to take the time to pause, reflect, and be philosophically creative. Perplexity, precisely because it alienates us from our habits and surroundings, allows us to achieve a cognitive distance from our philosophical assumptions (pp. 214–215).

As we worked to make sense of what was happening, we asked, who are we alongside the mothers and children? And who are they in relation to us? We were reminded as we inquired into our experience that morning of Addams (1902) who, in sustaining a humble attitude, honored and respected the perplexities that arose in the spaces between herself, a white woman of privilege and stability, and her immigrant neighbors, many of whom lived lives marked by experiences of poverty. Adams acknowledged the troubling perplexity that can occur when personal involvement in a set of circumstances creates bewilderment, and the usual ways of responding are no longer helpful in understanding the experience and concerns of others.

As we inquired into our experiences with the mothers and children, we began to understand how Addams’ (1902) practices were a kind of guide to what we were attempting to live out alongside them in our Friday morning gatherings and other occasions. As we worked to understand the celebratory party, we saw it as an occasion for the women to recreate their home places, to create a place for them to come together at an event, a wedding, that was part of all of their lives in Syria. It allowed them to make a place where they could be with other women and their children, to eat, drink, and dance, together. They were also intentional with us, inviting us into a symbolic staged event where we might come alongside in the living rather than only in their telling stories of their lives.

It is interesting to think that in their expression of their logic of place (Pratt, 2002) they are also resisting being subsumed into what their experiences alone in separate homes in Canada were beginning to become. They were not forsaking their Syrian homeplace but innovating to make it anew. In resisting, they were restoring, and with their relationship alongside us, renewing their place. They were in charge in this Friday morning space, and they could make something that mattered to them. They took charge of the space that was usually focused on providing what they needed, English lessons, resources, and child care. Perhaps what we experienced that morning of the wedding celebration was akin to what Nayeri (2019) referred to as “friendship, not salvation. They need the dignity of becoming an essential part of a society” (p. 338). The women’s intentional act of community building, drawing on domestic details, became visible, and we, as narrative inquirers, had to be willing to navigate unknown waters—to metaphorically travel to places and times in their lives we had not yet visited. We are reminded of Nayeri’s (2019) words: “You need no expertise to aim for curiosity. To care is enough” (p. 343). For Addams, avoiding perplexity by holding on to what one already knows are missed opportunities for learning. On that particular Friday morning, we saw these women anew and we learned something new, because we were willing to let go of the stories we knew and the stories we had been told—“allowing newcomers to affect [us] on [our] native soil, to change [us]” (Nayeri, 2019, p. 342).

## In the Midst of Silences: Not Turning Away

We could have, as researchers turned away that morning, seeing the celebrations as a time when the focus was not on what usually happened on Friday mornings: there was no language instruction, no chance to sit quietly and talk with the women; no opportunity to see the children at play. We did not turn away, for if we had, it would have constituted a profound silencing of the unfolding lives of the women and children, which they had claimed as a time to celebrate their lives in their new places in Edmonton. We could interpret that morning as a kind of silencing, as a resistance to English classes, as a resistance to what was constructed in the settlement sector as integration.

Remaining wakeful (Greene, 1995), we instead saw the celebration as an opening to see their lives in action, to see their living. The celebratory space allowed us to experience the domestic details up close and to see them engaged in acts of resistance to the Friday morning purposes of seeing them as refugees in need of learning English, of needing resources, of needing more skills to allow them to shape their lives in their new country. In resisting, they were reclaiming their agency. They were making something happen. Within their celebratory space, their voices drowned out the silences bestowed upon them by dominant narratives.

That day things changed for us, the connections felt different—a chance perhaps to experience more of living alongside in places that were strange to us. A glimpse into the perhaps domestic details of the women’s lives in Syria. They created the event for themselves, a time to feel “at home” in Canada but as we lived in the space with them they were anxious to tell us what they were doing.

Amidst the business, noise, and activities that morning was also a kind of silence. As researchers we were silent and were trying to make sense of what was happening. The children were watching their mothers with great intentions and with awe. Those who danced, moved their bodies to rhythms they had learned so long ago.

Thinking back, allowed us to reflect on narrative inquiry as lived in the midst of silences. There were many silences present on the mornings we came to be with the mothers and children. Silences that were created and amplified by dominant narratives. Previously, we (Blix et al., 2021) have written about silence as a quietness that allows a turning inward—it is a choice to be respected leading to a fuller dialogic understanding for one another. But we also talked of imposed silences, that are embedded in oppression and a loss of power. Yet we know that our experiences of silences are part of our lives and shaped by living alongside in the midst of many complex relationships. That morning, the silences that are marked by oppression and a way of silencing were not what we noticed. That morning, however, we noticed the kind of silence that allowed for awe and wonderment, that allowed for bodies and movements to be noticed, and perhaps the kind of silence that allows us to carry forward embodied narratives.

Elsewhere we have written about the importance of silence in narrative inquiry:

We believe that narrative inquiry is not necessarily about breaking silences. Narrative inquiry is also about honoring silences, and it is about practicing silence. As narrative inquirers, we must think carefully about which silences to break, which silences to protect, and how we practice silence. A first step in such a direction is being wakeful to the silences in and between people’s stories, to the silences in our own lives, and to the functions the silences may serve in both our own and others’ lives (Blix et al., 2021, p. 592).

In retrospect, we wonder about the possibilities that exist when we live alongside silences, pauses that are part of our everyday living. These perhaps are obscured when we primarily focus on telling, rather than living stories. Silences are also places of learning and unlearning. They are part of moments when we enter a room of unexpected celebrations. These are the silences that come to life when we are living in the midst.

## Conclusion

Our participation in the Friday morning gatherings with the mothers and their children was the beginning of a long-term commitment to create relationships. Addams (1902) claims that long-term everyday commitments are necessary due to the complexities inherent in working with people from diverse communities. While our opening field text gives a sense of our participation in the midst, we were hesitant about who we were at the outset in meetings with people who worked with the [newcomer cooperative organization]. We knew that our lives were very different from the mothers and children we imagined we would meet. We often wondered out loud if we would be able to understand even a fragment of the lives of refugees from Syria. Could we ever understand what living in the midst meant for them?

We carefully outlined our intentions and commitments and were met with many questions about how long would we come for? Who would attend? What would we do during the Friday morning gatherings? How could we introduce the research study in transparent and ethical ways? Were we looking for particular participants? There was excitement, but also hesitation. We were mindful to not be seen as, or to become, a burden.

Coming alongside the mothers and children through mutual engagement in the Friday morning English classes over many months made visible the ways we as researchers and potential participants were working on something together. Through long-term everyday commitment, a place of belonging for both researchers and mothers and children was coming into existence. It became a place of being in the midst of unfolding relationships and stories. There was a kind of rhythm that marked our time together, but there were also welcomed disruptions, like the enacted wedding. This rhythm and the disruptions helped us begin to see that lives, both those of research participants and researchers, are lived in the midst of dominant stories, domestic details, and silences. Attending to living in the midst allows us to experience and contemplate perplexities, it enables wonders to emerge amid a plurality of experiences. These experiences cannot be represented as fragments, as V experienced in the guest seminar. To understand narrative inquiry, we need to understand fragments as embedded in long-term relational engagement that often stretch over months and years. They are part of living in the midst of unfolding lives and relationships.
